# *N*-(2,4-Dinitro­phen­yl)-*N*′-(1-*p*-tolyl­ethyl­idene)hydrazine

**DOI:** 10.1107/S1600536809009957

**Published:** 2009-03-25

**Authors:** Reza Kia, Hoong-Kun Fun, Bijan Etemadi, Hadi Kargar

**Affiliations:** a X-ray Crystallography Unit, School of Physics, Universiti Sains Malaysia, 11800 USM, Penang, Malaysia; b Department of Earth Sciences, College of Sciences, Shiraz University, Shiraz, Iran; c Department of Chemistry, School of Science, Payame Noor University (PNU), Ardakan, Yazd, Iran

## Abstract

In the title mol­ecule, C_15_H_14_N_4_O_4_, the dihedral angle between the two benzene rings is 2.21 (7)°. An intra­molecular N—H⋯O hydrogen bond generates an *S*(6) ring motif. The mean planes of the *ortho*- and *para*-nitro groups make dihedral angles of 2.17 (17) and 2.05 (16)°, respectively, with the benzene ring to which they are attached. In the crystal structure, weak inter­molecular C—H⋯O hydrogen bonds generate *R*_2_^2^(7), *R*_2_^2^(13) and *R*_2_^1^(10) ring motifs, linking symmetry-related mol­ecules into extended chains along the *b* axis. In addition, there are inter­molecular C⋯C [3.332 (2)–3.343 (2) Å] contacts which are shorter than the sum of the van der Waals radii. The crystal structure is further stabilized by inter­molecular C—H⋯π and π–π stacking inter­actions [centroid–centroid distance = 3.8090 (9) Å].

## Related literature

For bond-length data, see: (Allen *et al.* 1987 ▶). For hydrogen-bond ring motifs, see: Bernstein *et al.* (1995 ▶). For related structures, see: Fun *et al.* (2009 ▶); Kia *et al.* (2009 ▶). For background information on 2,4-dinitro­phenyl­hydrazones, see: Cordis *et al.* (1998 ▶); Guillaumont & Nakamura (2000 ▶); Lamberton *et al.* (1974 ▶); Niknam *et al.* (2005 ▶); Raj & Kurup (2006 ▶); Zegota (1999 ▶); Zlotorzynska & Lai (1999 ▶); For the synthetic procedure, see: Okabe *et al.* (1993 ▶). For the stability of the temperature controller used for the data collection, see: Cosier & Glazer (1986 ▶).
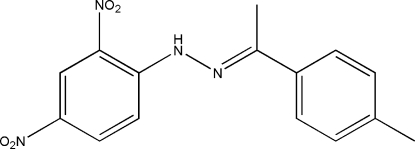


## Experimental

### 

#### Crystal data

C_15_H_14_N_4_O_4_
*M* *_r_* = 314.30
Monoclinic, 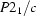

*a* = 7.6948 (1) Å
*b* = 14.9092 (3) Å
*c* = 12.5224 (2) Å
β = 91.778 (1)°
*V* = 1435.92 (4) Å^3^
*Z* = 4
Mo *K*α radiation
μ = 0.11 mm^−1^
*T* = 100 K
0.25 × 0.23 × 0.15 mm

#### Data collection

Bruker SMART APEXII CCD area-detector diffractometer
Absorption correction: multi-scan (**SADABS**; Bruker, 2005 ▶) *T*_min_ = 0.974, *T*_max_ = 0.984
16826 measured reflections
4211 independent reflections
3206 reflections with *I* > 2˘*I*)
*R*_int_ = 0.030

#### Refinement

*R*[*F* ^2^ > 2σ(*F* ^2^)] = 0.057
*wR*(*F* ^2^) = 0.143
*S* = 1.08
4211 reflections
214 parameters
H atoms treated by a mixture of independent and constrained refinement
Δρ_max_ = 0.42 e Å^−3^
Δρ_min_ = −0.26 e Å^−3^

### 

Data collection: *APEX2* (Bruker, 2005 ▶); cell refinement: *SAINT* (Bruker, 2005 ▶); data reduction: *SAINT*; program(s) used to solve structure: *SHELXTL* (Sheldrick, 2008 ▶); program(s) used to refine structure: *SHELXTL*; molecular graphics: *SHELXTL*; software used to prepare material for publication: *SHELXTL* and *PLATON* (Spek, 2009 ▶).

## Supplementary Material

Crystal structure: contains datablocks global, I. DOI: 10.1107/S1600536809009957/lh2788sup1.cif

Structure factors: contains datablocks I. DOI: 10.1107/S1600536809009957/lh2788Isup2.hkl

Additional supplementary materials:  crystallographic information; 3D view; checkCIF report

## Figures and Tables

**Table 1:** Hydrogen-bond geometry (Å, °)

*D*—H⋯*A*	*D*—H	H⋯*A*	*D*⋯*A*	*D*—H⋯*A*
N1—H1*N*1⋯O4	0.85 (2)	1.96 (2)	2.5966 (18)	131.3 (18)
C4—H4*A*⋯O4^i^	0.93	2.51	3.232 (2)	135
C5—H5*A*⋯O3^i^	0.93	2.55	3.4409 (19)	161
C9—H9*A*⋯O3^i^	0.93	2.41	3.295 (2)	158
C14—H14*C*⋯*Cg*1^ii^	0.96	2.68	3.5635 (17)	154

Symmetry codes: (i) 
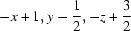
; (ii) 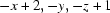. *Cg*1 is the centroid of the C8–C13 benzene ring.

**Fig. 1.**
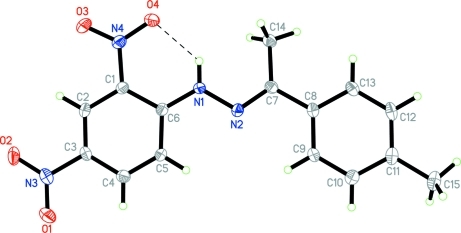
The molecular structure of the title compound, showing 50% probability displacement ellipsoids and the atomic numbering scheme. The dashed line indicates a hydrogen bond.

**Fig. 2.**
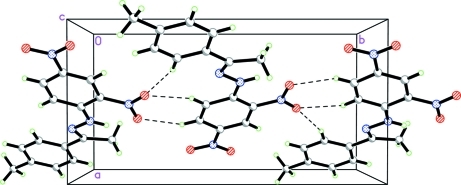
Part of the crystal structure of the title compound, viewed along the c-axis, showing molecules linked by weak intermolecular C—H···O interactions along the b-axis. Intermolecular interactions are shown as dashed lines.

## supplementary crystallographic information

### Comment

2,4-Dinitrophenylhydrazones play a more important role as stabilizers for the detection, characterization and protection of carbonyl groups than do phenylhydrazones (Niknam *et al.*, 2005). 2,4-Dinitrophenylhydrazone derivatives are widely used in various forms of analytical chemistry (Lamberton *et al.*, 1974; Zegota, 1999; Cordis *et al.*, 1998; Zlotorzynska & Lai, 1999) and are also used as dyes (Guillaumont & Nakamura, 2000). They are also found to have versatile coordinating abilities towards different metal ions (Raj & Kurup, 2006). In addition, some phenylhydrazone derivatives have been shown to be potentially DNA-damaging and mutagenic agents (Okabe *et al.,* 1993). The above information attracted our interest in the title compound and the the crystal structure is reported herein.

The bond lengths (Allen *et al.,* 1987) and angles in the title compound (Fig. 1) have normal values and are comparable to the related structures (Fun *et al.* 2009; Kia *et al.* 2009). An intramolecular N—H···O hydrogen bond generates a *S(6)* ring motif (Bernstein *et al.*, 1995). Weak intermolecular C—H···O hydrogen bonds generate *R_2_^2^(7)*, *R_2_^2^(13)* and *R_2_^1^(10)* ring motifs. These interactions link symmetry related molecules into extended chains along the *b* axis (Fig. 2). The molecule is approximately planar, with the maximum deviation from the mean plane of the molecule being 0.381 (2) Å for atom C14. The dihedral angle between the two benzene rings is 4.63 (1)°. Some interesting features of the crystal structure are the short intermolecular contacts of O2···O2^i^ [3.0319(170 Å; (i) -x, -y, 2 - z] and O2···N3^i^ [3.0513 (18) Å] and in addition the C2···C3^iii^ [3.332 (2) Å; (iii) 1 - x, -y, 2 - z ], C2···C12^iv^ [3.340 (2) Å; (iv) 1 - x, -y , 1 - z], C7···C13^ii^ [3.343 (2) Å] contacts which are shorter than the sum of the van der Waals radii. The crystal structure is further stabilized by intermolecular C—H···π and π-π interactions [*Cg*1···*Cg*2 = 3.8090 (9) Å; *Cg*1 and *Cg*2 are the centroids of the C8–C13 and C1–C6 benzene rings].

### Experimental

The title compound was synthesized based on the reported procedure (Okabe *et al.* 1993) except that 4-methyl-acetophenone (1 mmol) was used instead. Single crystals suitable for X-ray diffraction analysis were grown by slow evaporation of a saturated solution of the resulted compound in acetone.

### Refinement

The N-bound H atom was located from the difference Fourier map and refined freely; see, Table 1. The remaining H atoms were positined geometrically and constrained with a riding model approximation with C—H = 0.93–0.96 Å and U_iso_(H) = 1.2 or 1.5 U_eq_(C). A rotating group model was applied to the methyl groups.

### Crystal data

**Table:**

C_15_H_14_N_4_O_4_	*F*(000) = 656
*M**_r_* = 314.30	*D*_x_ = 1.454 Mg m^−^^3^
Monoclinic, *P*2_1_/*c*	Mo *K*α radiation, λ = 0.71073 Å
Hall symbol: -P 2ybc	Cell parameters from 5279 reflections
*a* = 7.6948 (1) Å	θ = 2.7–30.0°
*b* = 14.9092 (3) Å	µ = 0.11 mm^−^^1^
*c* = 12.5224 (2) Å	*T* = 100 K
β = 91.778 (1)°	Block, red
*V* = 1435.92 (4) Å^3^	0.25 × 0.23 × 0.15 mm
*Z* = 4	

### Data collection

**Table:**

Bruker SMART APEXII CCD area-detector diffractometer	4211 independent reflections
Radiation source: fine-focus sealed tube	3206 reflections with *I* > 2˘*I*)
graphite	*R*_int_ = 0.030
φ and ω scans	θ_max_ = 30.1°, θ_min_ = 2.1°
Absorption correction: multi-scan (*SADABS*; Bruker, 2005)	*h* = −10→10
*T*_min_ = 0.974, *T*_max_ = 0.984	*k* = −20→17
16826 measured reflections	*l* = −17→17

### Refinement

**Table:**

Refinement on *F*^2^	Primary atom site location: structure-invariant direct methods
Least-squares matrix: full	Secondary atom site location: difference Fourier map
*R*[*F*^2^ > 2σ(*F*^2^)] = 0.057	Hydrogen site location: inferred from neighbouring sites
*wR*(*F*^2^) = 0.143	H atoms treated by a mixture of independent and constrained refinement
*S* = 1.08	*w* = 1/[σ^2^(*F*_o_^2^) + (0.0631*P*)^2^ + 0.5547*P*] where *P* = (*F*_o_^2^ + 2*F*_c_^2^)/3
4211 reflections	(Δ/σ)_max_ = 0.001
214 parameters	Δρ_max_ = 0.42 e Å^−^^3^
0 restraints	Δρ_min_ = −0.26 e Å^−^^3^

### Special details

**Table:**

Experimental. The crystal was placed in the cold stream of an Oxford Cyrosystems Cobra open-flow nitrogen cryostat (Cosier & Glazer, 1986) operating at 100.0 (1)K.
Geometry. All esds (except the esd in the dihedral angle between two l.s. planes) are estimated using the full covariance matrix. The cell esds are taken into account individually in the estimation of esds in distances, angles and torsion angles; correlations between esds in cell parameters are only used when they are defined by crystal symmetry. An approximate (isotropic) treatment of cell esds is used for estimating esds involving l.s. planes.
Refinement. Refinement of F^2^ against ALL reflections. The weighted R-factor wR and goodness of fit S are based on F^2^, conventional R-factors R are based on F, with F set to zero for negative F^2^. The threshold expression of F^2^ > 2sigma(F^2^) is used only for calculating R-factors(gt) etc. and is not relevant to the choice of reflections for refinement. R-factors based on F^2^ are statistically about twice as large as those based on F, and R- factors based on ALL data will be even larger.

### Fractional atomic coordinates and isotropic or equivalent isotropic displacement parameters (Å^2^)

**Table:**

	*x*	*y*	*z*	*U*_iso_*/*U*_eq_	
O1	0.17776 (17)	0.87567 (9)	1.02191 (10)	0.0328 (3)	
O2	0.16471 (16)	1.01618 (9)	1.06678 (9)	0.0279 (3)	
O3	0.45972 (17)	1.23529 (8)	0.87222 (10)	0.0312 (3)	
O4	0.60603 (15)	1.20508 (8)	0.73223 (9)	0.0260 (3)	
N1	0.62455 (17)	1.04202 (9)	0.66064 (10)	0.0176 (3)	
N2	0.67704 (16)	0.97591 (9)	0.59199 (10)	0.0175 (3)	
N3	0.21243 (18)	0.95518 (10)	1.00792 (10)	0.0228 (3)	
N4	0.51408 (17)	1.18141 (9)	0.80690 (10)	0.0210 (3)	
C1	0.47019 (19)	1.08784 (10)	0.81806 (11)	0.0167 (3)	
C2	0.36357 (19)	1.06601 (10)	0.90212 (11)	0.0178 (3)	
H2A	0.3226	1.1105	0.9469	0.021*	
C3	0.3200 (2)	0.97796 (11)	0.91782 (11)	0.0180 (3)	
C4	0.3812 (2)	0.90980 (11)	0.85198 (11)	0.0191 (3)	
H4A	0.3528	0.8503	0.8653	0.023*	
C5	0.4829 (2)	0.93102 (10)	0.76795 (11)	0.0177 (3)	
H5A	0.5221	0.8854	0.7241	0.021*	
C6	0.52995 (19)	1.02107 (10)	0.74630 (11)	0.0159 (3)	
C7	0.74308 (19)	1.00482 (10)	0.50440 (11)	0.0169 (3)	
C8	0.80349 (19)	0.93424 (10)	0.43032 (11)	0.0172 (3)	
C9	0.7621 (2)	0.84429 (11)	0.44813 (12)	0.0208 (3)	
H9A	0.6965	0.8292	0.5066	0.025*	
C10	0.8168 (2)	0.77741 (11)	0.38063 (13)	0.0247 (4)	
H10A	0.7869	0.7182	0.3943	0.030*	
C11	0.9165 (2)	0.79729 (12)	0.29186 (12)	0.0223 (3)	
C12	0.9582 (2)	0.88663 (12)	0.27404 (12)	0.0223 (3)	
H12A	1.0245	0.9015	0.2158	0.027*	
C13	0.9029 (2)	0.95450 (11)	0.34177 (11)	0.0198 (3)	
H13A	0.9323	1.0138	0.3279	0.024*	
C14	0.7563 (2)	1.10272 (11)	0.47653 (12)	0.0208 (3)	
H14A	0.6455	1.1310	0.4853	0.031*	
H14B	0.7899	1.1088	0.4037	0.031*	
H14C	0.8418	1.1308	0.5229	0.031*	
C15	0.9752 (2)	0.72351 (13)	0.21878 (14)	0.0298 (4)	
H15A	0.8783	0.6855	0.2000	0.045*	
H15B	1.0642	0.6887	0.2547	0.045*	
H15C	1.0205	0.7494	0.1552	0.045*	
H1N1	0.652 (3)	1.0963 (15)	0.6502 (15)	0.027 (5)*	

### Atomic displacement parameters (Å^2^)

**Table:**

	*U*^11^	*U*^22^	*U*^33^	*U*^12^	*U*^13^	*U*^23^
O1	0.0401 (8)	0.0280 (7)	0.0311 (6)	−0.0030 (6)	0.0136 (6)	0.0070 (5)
O2	0.0299 (6)	0.0348 (7)	0.0195 (5)	0.0014 (5)	0.0090 (5)	−0.0053 (5)
O3	0.0426 (8)	0.0173 (6)	0.0344 (7)	0.0007 (5)	0.0135 (6)	−0.0079 (5)
O4	0.0302 (6)	0.0173 (6)	0.0312 (6)	−0.0028 (5)	0.0114 (5)	0.0002 (5)
N1	0.0208 (6)	0.0129 (6)	0.0196 (6)	−0.0009 (5)	0.0067 (5)	−0.0008 (5)
N2	0.0175 (6)	0.0168 (6)	0.0183 (6)	0.0006 (5)	0.0035 (5)	−0.0022 (5)
N3	0.0218 (7)	0.0282 (8)	0.0184 (6)	0.0000 (6)	0.0030 (5)	0.0035 (5)
N4	0.0228 (7)	0.0164 (7)	0.0240 (6)	0.0008 (5)	0.0030 (5)	−0.0022 (5)
C1	0.0179 (7)	0.0147 (7)	0.0176 (6)	−0.0001 (6)	0.0008 (5)	−0.0014 (5)
C2	0.0180 (7)	0.0199 (8)	0.0157 (6)	0.0028 (6)	0.0021 (5)	−0.0035 (5)
C3	0.0185 (7)	0.0218 (8)	0.0139 (6)	−0.0007 (6)	0.0025 (5)	0.0014 (5)
C4	0.0208 (7)	0.0174 (7)	0.0190 (7)	−0.0013 (6)	−0.0001 (6)	0.0016 (6)
C5	0.0217 (8)	0.0155 (7)	0.0162 (6)	0.0007 (6)	0.0022 (6)	−0.0027 (5)
C6	0.0156 (7)	0.0163 (7)	0.0158 (6)	−0.0002 (5)	0.0007 (5)	−0.0013 (5)
C7	0.0150 (7)	0.0181 (7)	0.0176 (6)	−0.0006 (5)	0.0005 (5)	0.0013 (5)
C8	0.0152 (7)	0.0214 (8)	0.0150 (6)	0.0017 (6)	0.0014 (5)	0.0004 (6)
C9	0.0229 (8)	0.0210 (8)	0.0190 (7)	−0.0007 (6)	0.0076 (6)	0.0005 (6)
C10	0.0267 (8)	0.0218 (8)	0.0260 (8)	−0.0009 (7)	0.0058 (7)	−0.0026 (6)
C11	0.0188 (7)	0.0300 (9)	0.0182 (7)	0.0033 (6)	0.0012 (6)	−0.0040 (6)
C12	0.0186 (7)	0.0345 (9)	0.0140 (6)	0.0027 (7)	0.0035 (6)	0.0017 (6)
C13	0.0187 (7)	0.0237 (8)	0.0169 (6)	−0.0001 (6)	0.0015 (6)	0.0036 (6)
C14	0.0238 (8)	0.0187 (8)	0.0200 (7)	0.0005 (6)	0.0031 (6)	0.0041 (6)
C15	0.0267 (9)	0.0357 (10)	0.0270 (8)	0.0029 (7)	0.0046 (7)	−0.0097 (7)

### Geometric parameters (Å, °)

**Table:**

O1—N3	1.2288 (19)	C7—C8	1.487 (2)
O2—N3	1.2336 (18)	C7—C14	1.505 (2)
O3—N4	1.2291 (16)	C8—C9	1.398 (2)
O4—N4	1.2412 (16)	C8—C13	1.3992 (19)
N1—C6	1.3515 (18)	C9—C10	1.381 (2)
N1—N2	1.3766 (17)	C9—H9A	0.9300
N1—H1N1	0.85 (2)	C10—C11	1.402 (2)
N2—C7	1.2968 (18)	C10—H10A	0.9300
N3—C3	1.4598 (18)	C11—C12	1.390 (2)
N4—C1	1.443 (2)	C11—C15	1.509 (2)
C1—C2	1.3931 (19)	C12—C13	1.395 (2)
C1—C6	1.4267 (19)	C12—H12A	0.9300
C2—C3	1.371 (2)	C13—H13A	0.9300
C2—H2A	0.9300	C14—H14A	0.9600
C3—C4	1.400 (2)	C14—H14B	0.9600
C4—C5	1.368 (2)	C14—H14C	0.9600
C4—H4A	0.9300	C15—H15A	0.9600
C5—C6	1.419 (2)	C15—H15B	0.9600
C5—H5A	0.9300	C15—H15C	0.9600
			
C6—N1—N2	120.39 (13)	C9—C8—C13	117.79 (13)
C6—N1—H1N1	118.9 (13)	C9—C8—C7	120.15 (12)
N2—N1—H1N1	120.7 (13)	C13—C8—C7	122.06 (14)
C7—N2—N1	114.86 (13)	C10—C9—C8	121.31 (13)
O1—N3—O2	123.78 (13)	C10—C9—H9A	119.3
O1—N3—C3	117.74 (13)	C8—C9—H9A	119.3
O2—N3—C3	118.47 (13)	C9—C10—C11	121.15 (16)
O3—N4—O4	121.96 (13)	C9—C10—H10A	119.4
O3—N4—C1	118.81 (12)	C11—C10—H10A	119.4
O4—N4—C1	119.23 (12)	C12—C11—C10	117.71 (14)
C2—C1—C6	121.45 (14)	C12—C11—C15	121.74 (14)
C2—C1—N4	116.36 (13)	C10—C11—C15	120.55 (16)
C6—C1—N4	122.18 (12)	C11—C12—C13	121.38 (13)
C3—C2—C1	118.99 (13)	C11—C12—H12A	119.3
C3—C2—H2A	120.5	C13—C12—H12A	119.3
C1—C2—H2A	120.5	C12—C13—C8	120.65 (15)
C2—C3—C4	121.51 (13)	C12—C13—H13A	119.7
C2—C3—N3	118.74 (13)	C8—C13—H13A	119.7
C4—C3—N3	119.71 (14)	C7—C14—H14A	109.5
C5—C4—C3	119.76 (14)	C7—C14—H14B	109.5
C5—C4—H4A	120.1	H14A—C14—H14B	109.5
C3—C4—H4A	120.1	C7—C14—H14C	109.5
C4—C5—C6	121.41 (13)	H14A—C14—H14C	109.5
C4—C5—H5A	119.3	H14B—C14—H14C	109.5
C6—C5—H5A	119.3	C11—C15—H15A	109.5
N1—C6—C5	121.14 (13)	C11—C15—H15B	109.5
N1—C6—C1	122.05 (14)	H15A—C15—H15B	109.5
C5—C6—C1	116.80 (12)	C11—C15—H15C	109.5
N2—C7—C8	115.51 (13)	H15A—C15—H15C	109.5
N2—C7—C14	123.34 (13)	H15B—C15—H15C	109.5
C8—C7—C14	121.14 (12)		
			
C6—N1—N2—C7	169.80 (14)	C2—C1—C6—N1	175.87 (15)
O3—N4—C1—C2	1.7 (2)	N4—C1—C6—N1	−3.2 (2)
O4—N4—C1—C2	−178.66 (14)	C2—C1—C6—C5	−3.1 (2)
O3—N4—C1—C6	−179.24 (15)	N4—C1—C6—C5	177.86 (14)
O4—N4—C1—C6	0.4 (2)	N1—N2—C7—C8	178.89 (13)
C6—C1—C2—C3	2.0 (2)	N1—N2—C7—C14	−2.2 (2)
N4—C1—C2—C3	−178.88 (14)	N2—C7—C8—C9	10.5 (2)
C1—C2—C3—C4	0.5 (2)	C14—C7—C8—C9	−168.44 (14)
C1—C2—C3—N3	178.22 (13)	N2—C7—C8—C13	−169.26 (14)
O1—N3—C3—C2	−178.53 (15)	C14—C7—C8—C13	11.8 (2)
O2—N3—C3—C2	0.0 (2)	C13—C8—C9—C10	−0.2 (2)
O1—N3—C3—C4	−0.8 (2)	C7—C8—C9—C10	−179.92 (15)
O2—N3—C3—C4	177.71 (15)	C8—C9—C10—C11	0.2 (3)
C2—C3—C4—C5	−1.9 (2)	C9—C10—C11—C12	−0.1 (2)
N3—C3—C4—C5	−179.57 (14)	C9—C10—C11—C15	−179.98 (16)
C3—C4—C5—C6	0.7 (2)	C10—C11—C12—C13	−0.2 (2)
N2—N1—C6—C5	0.2 (2)	C15—C11—C12—C13	179.76 (15)
N2—N1—C6—C1	−178.72 (14)	C11—C12—C13—C8	0.2 (2)
C4—C5—C6—N1	−177.27 (15)	C9—C8—C13—C12	0.0 (2)
C4—C5—C6—C1	1.7 (2)	C7—C8—C13—C12	179.69 (14)

### Hydrogen-bond geometry (Å, °)

**Table:**

*D*—H···*A*	*D*—H	H···*A*	*D*···*A*	*D*—H···*A*
N1—H1N1···O4	0.85 (2)	1.96 (2)	2.5966 (18)	131.3 (18)
C4—H4A···O4^i^	0.93	2.51	3.232 (2)	135
C5—H5A···O3^i^	0.93	2.55	3.4409 (19)	161
C9—H9A···O3^i^	0.93	2.41	3.295 (2)	158
C14—H14C···Cg1^ii^	0.96	2.68	3.5635 (17)	154

Symmetry codes: (i) −*x*+1, *y*−1/2, −*z*+3/2; (ii) −*x*+2, −*y*, −*z*+1.
